# How Should the Teeth Be Cleansed

**Published:** 1900-11-15

**Authors:** C. Rose


### DENTAL REGISTER.

Vol. LIV. November 15, 1900. No. 11.

### HOW SHOULD THE TEETH BE CLEANSED.

BY C. ROSE.

Extract from a Paper in *Dental Cosmos*.

The mouth is first to be rinsed, in order to remove coarse, loosely adherent remains of food. The brush is next moistened; some mouth-wash is held in the mouth; the edges of the front teeth are brought together, and their external surface brushed up and down. The external surfaces of the molars are also to be brushed, especially in this way, from above downward.

The mouth is next to be widely opened, and the grinding-surfaces of the bicuspids and molars are to be brushed from before backward and from left to right. The inner surfaces, as well as the interstices and angles of improperly implanted teeth, are then to be cleansed with the pointed cones.

The gums are, at the same time, to be freed from any deposits. The brush is then to be turned outward, and the mucous membrane of the cheek and the folds between them and the jaws cleansed. Finally, the tongue is brushed.

The dead superficial epithelial cells of the tongue, together with mucus, saliva, and bits of food, constitute the so-called "coating" of the tongue. Many persons use "tongue scrapers" for the removal of this coating. The advantages of these tools are as doubtful as they are unappetizing. The coating can be much more thoroughly and safely removed by the aid of the brush. Any tendency to nausea which may be provoked in the beginning quickly ceases.

It is of the utmost importance to accustom one's self to retain a mouthful of water in the mouth while using the brush, in order that the product of brushing may be immediately taken up by the fluid, and not simply pushed hither and thither, as is the case when the teeth are brushed with the mouth empty.

If while brushing the teeth air is slowly inspired through the mouth, the head properly inclined, and a suitable small brush employed, the advantages of the above-mentioned process will soon be apparent.

The narrow interstices between the teeth alone remain, in which no brush can penetrate. Tooth-picks serve to cleanse these spaces; soft quills are the best. Nickel cases for carrying the quills in the pocket can be purchased in the shops.

Toothpicks made of thin pieces of tortoise shell or wood are less desirable, being too thick. Metal should be avoided by all means. It readily injures the teeth and gums.

The pick is inserted in an interstice between two teeth and gently rotated, scraping the bits first from one and then from the adjacent surface. Excessive and useless picking should be carefully avoided.

Exceptionally, the thin quill can not be inserted between two closely placed teeth. In such cases the space is to be cleansed with a piece of waxed silk thread or stretched rubber.

Finally, the mouth is to be carefully rinsed several times, the fluid being forced back and forth in the intervals between the teeth by suction movements of the cheeks and tongue. Shaking the head, as is frequently done while rinsing the mouth, is a useless procedure.

After thoroughly cleansing the brush of any adherent matters by washing in clean water or an antiseptic solution, the fluid is removed by striking the brush against a resisting body. It is then hung up in the air to dry rapidly. It would appear more proper to place the brush after cleansing in an antiseptic mouth-wash, and to leave it therein until again required.

The ordinary porcelain cups for holding tooth-brushes should never be employed for that purpose; they readily cause the bristles to rot.

Unhappily, the so-called "family tooth-brushes" have not yet been altogether consigned to the region of fable. Servants also occasionally use their masters' or mistresses' brushes on the sly. To avoid these dangers, it is advisable to provide a brush not only for each member of the family, but also for the servants.

Hygienic measures are practiced in proportion to the pleasant sensations they call forth. The mechanical cleansing of the mouth and teeth is not a process calculated to excite feelings of pleasure *per se*. Accordingly fragrant and tasty mouth and tooth-washes have been recommended for centuries, instead of ordinary water for rinsing the mouth. The pleasant odor of these washes serves at the same time to conceal the fetid breath of such as require it.

After the bacteria had been recognized as the cause of diseases of the teeth and mouth, agents destructive of these organisms were naturally added to the mouth-washes. Many dentists even attempted to sterilize the entire oral cavity, with the idea of preventing caries in this way. The most powerful antiseptics were recommended for rinsing the mouth, without taking into consideration their dangerous properties. Trade soon came into this field, and the public was informed that the use of antiseptic mouth-washes was able of itself to prevent diseases of the teeth. If we consider that, in addition to the few really useful preparations, numerous worthless, and some even dangerous ones, all under the title of "antiseptic mouth-washes," were foisted upon the market, it will not seem strange that a justifiable opposition should have made itself felt. The brush! The wash! These were the shibboleths of this merry war. Finally a truce was arranged. Competent specialists are now agreed that while mechanical cleansing must ever remain the groundwork of all rational care of the teeth, suitable antiseptic mouth-washes are commendable.

A suitable mouth-wash should possess the following properties:

1. Absolutely uninjurious (a) to the teeth, (b) to the oral mucous membrane, (c) to the organism as a whole.

2. Effective antisepsis.

3. Pleasant taste and odor.

These various properties are naturally rarely found in combination, and yet each one is of equal importance. A mouth-wash which has a disgusting taste is as ineffective as one which has no germicidal action. The great mass of the public will never be induced to practice a method of oral hygiene which involves an ill-tasting mouth-wash. It is, of course, of the greatest importance to insist upon absolute safety. The importance of oral antisepsis is not so great that we are justified in assuming the slightest risk.

A mouth-wash may have the following untoward effects:

1. Toxicity to the organism as a whole.

2. Cauterization of the oral mucous membrane.

3. Decalcification of the teeth.

An agent which cauterizes the mucous membrane, such as corrosive sublimate, formaldehyd, kosmin, soap, etc., is at least as injurious as one which decalcifies the teeth. In consequence of the recurrent cauterizations, the mucous membrane finally inflames, forming an excellent field for the culture of pathogenic organisms.

As opposed to such effects, a suitable antiseptic mouth-wash must favor recovery of an inflamed mucous membrane, which is so often present to a mild degree.

During the course of the last few years I have made extensive experiments upon the efficiency of the ordinary mouth-washes (Rose, "The Vegetable Parasites of the Mouth and Their Destruction," Reports of the Society of Morphology and Physiology of Munich, 1889). I found that "odol," in spite of the exaggerated claims made for it in advertisements, must be regarded as the best known mouth wash, in respect to safety, antiseptic power, and pleasant taste. Unfortunately, the maker refuses to reveal the chemical composition of his antiseptic. Among mouth-washes whose composition is known, that of Miller is to be commended. It is as follows:

| | | |
|---|---|---|
| ℞. | Acidi benzoici . . . . . | 3. |
| | Tinct. ratanhæ . . . . . | 15. |
| | Alcohol . . . . . . . | 100. |
| | Ol. menthæ . . . . . . | 0.75 |

*Sig.*—One teaspoonful to a small wineglass of water.

The taste of this compound is not entirely unobjectionable. A pinch of table salt added to a lukewarm solution of odol or Miller's mouth-wash materially increases the germicidal effect of the remedy, causing it to assume the properties of 0.7 percent normal solution of sodium chlorid, which, as is well known, possesses decided antibacterial properties. Such a weak solution of table salt (equivalent to the quantity borne upon the point of a knife to a wineglass or a thimbleful to an ordinary tumbler of lukewarm water) is to be recommended for persons seriously ill, little children, and those of limited means.

The ethereal oils present in all the mouth-washes of the shops give rise to an eruption (eczema) at the angles of the lips in many persons possessed of a rather delicate skin. Individuals predisposed to such eruptions should confine themselves to normal salt solution as a mouth-wash.

One of the most important prerequisites for the successful care of the teeth is a healthy and firm state of the mucous membrane. The best remedy for the diseased membrane is a solution of fifty to sixty percent alcohol. Rinsing can not, of course, be performed with such a solution, but we must be content to dip the brush into the alcohol before using. Excess in the use of alcohol is to be carefully avoided. The remedy is not suitable for daily use. Alcohol is like a whip, it fails of its purpose if too frequently used. Excessive application of alcohol in the mouth causes shrinking of the gums.

We come finally to the last series of measures directed to the care of the teeth. Their great importance has been largely underestimated during the last decennials. Tooth-powders were originally meant for beautifiers alone. They were intended to remove deposits which could not be rubbed off by the brush alone, so that the fine natural color of the teeth might show.

Vanity alone causes a good many persons to care for their teeth. They like to have their well-polished teeth admired. Who could blame the hygienist if he tried to enlist this passion in his service? If for no other reason, the use of harmless powders is to be recommended.

The principal constituent of almost all tooth-powders in use is carbonate of calcium or magnesium. Both of these compounds exercise, beside their mechanical effect, a decided chemical action, in that they neutralize the dangerous oral acids, the products of the caries bacilli. Thus, while antiseptic mouth-washes directly destroy the germs, suitable tooth-powders destroy their products. Stronger alkaline solutions can not be used to neutralize the oral acids; they cauterize the mucous membrane. "Dental soap" is, for the same reason, to be employed with great caution. The most perfect soaps give off free alkali in the presence of well water containing lime. He who insists upon using soap should at least use distilled water as a mouth-wash.

"Spirits of soap" may be occasionally employed by the dentist for the rapid cleansing of dirty mouths. The agent is entirely unsuitable for daily use. The entire oral epithelium strips off in long shreds after the use of the spirits, and the mucous membrane finally inflames.

"Tooth-pastes" are a mixture of powder and soap, with the addition of glycerin or a similar substance. They have, as a rule, the same disadvantages as the soap.

More gritty substances, like cigar ash, powdered pumice stone, etc., are not to be used; they are destructive of the enamel. We must, above all, be on our guard against all tooth-powders and pastes which rapidly give a glistening polish. They contain free acids which dissolve the enamel.

Antiseptic substances have been added to some tooth-powders. They are of no value, for the reason that the antiseptics can hardly undergo perfect solution during the short time of their presence in the mouth.

Vegetable powders, such as orris root, etc., which are often added to powder to make them more tasty, are to be avoided; they smear the teeth instead of cleansing them.

A suitable tooth-powder should consist principally of powdered calcium or magnesium carbonates, to which an ethereal oil may be added to improve the taste.

Many dentists entertain the opinion that the ordinary insoluble tooth-powders have the disadvantage of forming small masses with the remains of the food, and thus come to lodge in the interstices between the teeth. This is only possible when the cleansing is inadequate. But in such cases the mixture of the powder with the food possesses an added advantage. As soon as acids have been formed from the food, the alkali is there to antagonize the ill effects of the acid.

The fear that tooth-powder may favor the deposit of tartar is unfounded if the cleansing is thorough. Stimulated by the excessive fear of hypothetical disadvantages of the calcium powders, the attempt has been made during the last few years to prepare "soluble powders." These consist principally of bicarbonate of soda and borax. I have myself made many experiments for many years with the soluble carbonates, but have come to abandon them entirely. The salts are not gritty enough; their unpleasant taste can hardly be concealed, and is unbearable for any length of time.

The care of the mouth in nurslings requires a brief particular consideration. The mouth must be cared for even in infancy, especially during the time of the eruption of the teeth. Mothers and nurses usually wind a wet linen rag around their finger, which is then stuck in the child's mouth. Adequate cleansing of the mouth can not be attained in this crude fashion. The delicate mucous membrane is frequently injured. A small, soft tooth brush, or a pledget of cotton upon a stick, is less injurious. A suitable small syringe would seem most appropriate for cleansing the mouth, while the infant's nostrils are closed by the fingers and the head inclined forward, so that the water (normal salt solution) shall return immediately.

The dentist is very frequently asked, "How often should the teeth be cleansed?" Persons with teeth imperfectly developed will do well to cleanse them after each meal; above all after eating pastry, farinaceous sweets, or confections. The mouth is especially to be looked after while undergoing the "grape cure," after eating acid food, during pregnancy, affections of the stomach accompanied by acid eructations, and in the course of all serious diseases.

It behooves every physician who desires the gratitude of his bed-ridden patients to provide for the regular care of their mouths. The oral epithelium is shed to a considerable extent in all febrile affections. In consequence of the increased temperature, decomposition takes place much more rapidly.

Simple rinsing of the mouth with a weak solution of salt gives great relief in such cases. Patients too ill to raise their heads may be enabled to rinse their mouths by means of the medicine cup. Water is introduced into the mouth through the spout, and ejected in the same way after rinsing.

Whoever is prevented by external circumstances from cleansing his teeth more than once a day should do this in the evening, before retiring. Caries progresses most rapidly during sleep, when the cheeks and tongue are in repose and no current of alkalin saliva is present to inhibit the destructive action of the oral acids. Whoever cleanses the teeth in the morning only is covering the well after the child has fallen in.

